# Dissipation Behavior, Residue, and Risk Assessment of Benziothiazolinone in Apples

**DOI:** 10.3390/ijerph18094478

**Published:** 2021-04-23

**Authors:** Yida Chai, Rong Liu, Wei He, Fuliu Xu, Zenglong Chen, Li Li, Wei Li, Longfei Yuan

**Affiliations:** 1 State Key Laboratory of Integrated Management of Pest Insects and Rodents, Institute of Zoology, Chinese Academy of Sciences, Beijing 100101, China; crise9898665@icloud.com (Y.C.); chenzenglong@ioz.ac.cn (Z.C.); lili2008@ioz.ac.cn (L.L.); liw@ioz.ac.cn (W.L.); 2 School of Agriculture, Yangtze University, Jingzhou 434025, China; 3 Institute of Crop Sciences, Chinese Academy of Agricultural Sciences, Beijing 100081, China; liurong@caas.cn; 4 School of Water Resources and Environment, China University of Geosciences, Beijing 100083, China; 5 College of Urban and Environmental Sciences, Peking University, Beijing 100871, China; xufl@urban.pku.edu.cn

**Keywords:** benziothiazolinone, dissipation behavior, residue, dietary risk assessment, apple

## Abstract

Benziothiazolinone is the first independently developed fungicide in China. It has been used to effectively control fungal diseases in a variety of fruits, vegetables, and crops. In this study, the degradation behavior and final residue of benziothiazolinone in apples is discussed, and the dietary risk to consumers was evaluated. High-performance liquid chromatography–tandem mass spectrometry (LC-MS/MS) was used to determine benziothiazolinone residues in apple samples from eight different regions of China. The average recovery of benziothiazolinone in apples was 85.5–100.2%, and the relative standard deviation (RSD) was 0.8–14.9%. The limits of the method of quantification of benziothiazolinone in apples was 0.01 mg/kg. Under good agricultural practices (GAP) conditions, the final residues of benziothiazolinone in apples were below 0.01 mg/kg, lower than the maximum residual limit (MRL) of China. Although the degradation half-lives of benziothiazolinone were 23.9 d–33.0 d, the risk quotient (RQ) of benziothiazolinone was 15.5% by calculating the national estimated daily intake and comparing it with the acceptable daily intake. These results suggested that under GAP conditions, the intake of benziothiazolinone from apples exhibits an acceptably low health risk on consumers.

## 1. Introduction

China is the largest apple producer and consumer in the world, accounting for about half of the world’s apple production. Apple has become an important part of Chinese diet [1]. Apple ring rot is one of the main apple diseases in China, which is widely distributed in most apple planting areas in China, resulting in a serious reduction in apple production [2]. It is necessary to apply high-efficiency and low-toxicity fungicides to prevent and treat apple ring disease.

**Figure 1:**
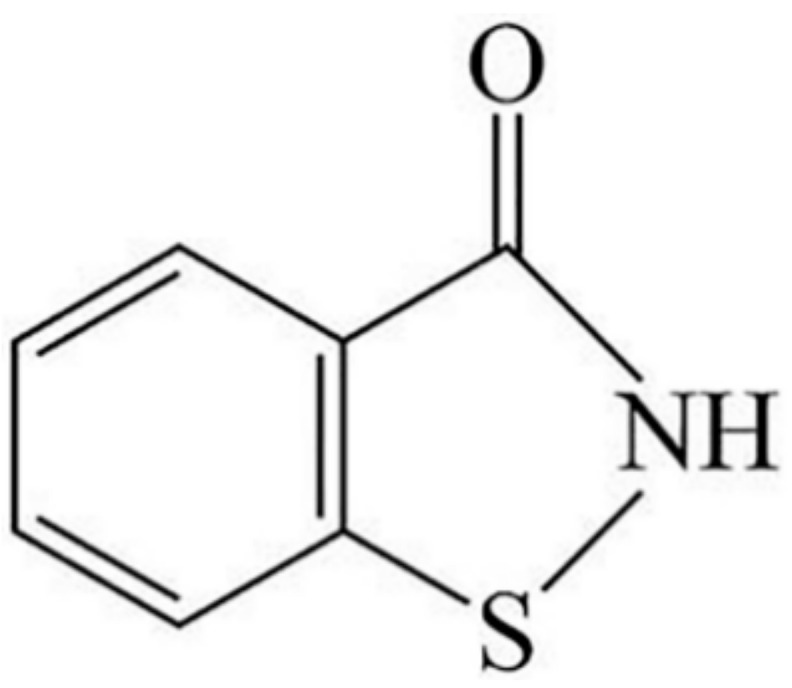
Structure of benziothiazolinone.

Benziothiazolinone (1,2-Benzisothiazol-3-one) is the first independently developed fungicide in China. Its chemical structure is shown in Figure 1. As an effective fungicide, it is mainly used in industrial sterilization, antisepsis, and mildew prevention in developed countries, such as Europe and the United States, while it has been used to control fungal diseases in China [3,4,5]. The registered crops include rice, wheat, cucumber, apple, and pear, and so on. The acute oral LD50 of benziothiazolinone is 1100 mg/kg, which is a low toxicity compound. But up to now, only China has set the maximum residual limit (MRL) of benziothiazolinone in apples as 0.05 mg/kg [6], and other countries, including European countries and the United States, have not yet set any MRL standards of benziothiazolinone. The toxicity of benziothiazolinone showed that the allowable daily intake (ADI) value of benziothiazolinone was 0.017 mg/kg bw [6]. Thus, in order to avoid the potential harm of benziothiazolinone to public health, it is necessary to conduct adequate research on the dissipation and residues of benziothiazolinone in apple and carry out an effective dietary risk assessment.

Up to now, many studies have reported the residue analysis methods of benziothiazolinone in different crops. Yu et al. [3] and Zhao et al. [7] used LC-UV to establish an analytical method for the determination of benziothiazolinone residues in cucumber and wheat, respectively. Su et al. [8] and Liu et al. [9] developed an improved QuEChERS pretreatment method combined with liquid chromatography tandem mass spectrometry (LC-MS/MS) to establish a method for the determination of benziothiazolinone residues in fruits and vegetables and their products. However, there is no report on dissipation behavior, residue, and risk assessment of benziothiazolinone residues in apple by LC-MS/MS.

In this study, during the research cycle, the residues of benziothiazolinone in apples from eight representative locations in China were measured. The objective of this study is to establish an accurate, simple and sensitive method for the determination of benziothiazolinone in apples by LC-MS/MS as well as to evaluate the dietary intake risk of benziothiazolinone based on the final residues and toxicology data. This study could provide guidance for dietary risk assessment of benziothiazolinone residues in apple and provide an effective analytical method for the determination of benziothiazolinone in apple samples.

## 2. Materials and Methods

### 2.1. Chemicals and Reagents

Benziothiazolinone standard (purity 99.57%) was purchased from Dr. Ehrenstorfer Gmbh (Augsburg, Germany). HPLC-grade acetonitrile was obtained from Fisher Chemical Co., Ltd. (Waltham, MA, USA). Analytical grade sodium chloride (NaCl), anhydrous magnesium sulfate (MgSO_4_), and formic acid were obtained from Beijing Tongguang Fine Chemical Co., Ltd. and CNW Technologies GmbH, respectively. A 2 mL centrifuge tube with 50 mg C_18_, primary secondary amine (PSA) and graphitized carbon black (GCB) adsorbent pre-installed was provided by ANPEL Laboratory Technologies Inc. (Shanghai, China), and a syringe filter (nylon, 0.22 μm) was purchased from Bonna-Agela Technologies Co., Ltd. (Tianjin, China).

### 2.2. Extraction and Purification Process of Samples

Homogenized apple samples (10 g) were weighed and placed into a 50 mL centrifuge tube. Five milliliters of ultrapure water and 10 mL of acetonitrile were added and shaken for 5 min in a high-throughput tissue grinder and then worked in an ultrasonic cleaning tank at 50 kHz frequency for 20 min. Six grams of NaCl and 4 g anhydrous MgSO_4_ were added into homogenized samples for phase separation and shaken for 1 min immediately and then centrifuged for 5 min at 4000 rpm. After standing and layering, 1 mL upper acetonitrile phase was transferred to a 2-mL centrifuge tube containing 50 mg C_18_ with a vortex of 1 min. After that, the supernatant was filtered through a 0.22 μm nylon syringe filter and transferred into an autosampler vial for LC-MS/MS analysis.

### 2.3. Chromatography Conditions

Benziothiazolinone was analyzed by ultra-performance liquid chromatography (Waters ACQUITY UPLC H-Class) and tandem triple quadrupole mass spectrometry (Xevo TQD) with electrospray ionization (ESI) source operated in positive ion mode (ESI+). An ACQUITY UPLC BEH C18 Column (50 mm × 2.1 mm, 1.7 μm) was used to separate the target compound with the temperature of 30 °C. The mobile phases were 0.05% (*v*/*v*) formic acid aqueous solution (A) and methanol (B). The gradient elution conditions are shown in Table S1. The flow rate was 0.40 mL/min, and the sample injection volume was 3 μL. The gas temperature was set at 500 °C, with a gas flow rate of 1000 L/h for MS detection working conditions. The ion source temperature was 150 °C, and the capillary voltages were controlled at 3500 V under positive ion detection mode. Analytes were determined in multiple reaction monitoring (MRM) mode. Under the operating conditions above, benziothiazolinone was quantified based on the acquisition parameters listed in Table S2.

### 2.4. Method Validation

The standard samples of benziothiazolinone were accurately weighed, dissolved in acetonitrile, and diluted step by step to form a series of standard solutions. Then the standard solution of benziothiazolinone was spiked to the blank sample of apple at appropriate concentrations of 0.01, 0.05, 1.0 mg/kg. Five parallel treatments were performed at each spiked level. These samples were pretreated and detected following the procedure as mentioned previously above to study the accuracy and precision of this method.

The linearity was studied using a standard solution and matrix-matched calibration by analyzing standard samples of 0.010, 0.025, 0.050, 0.10, 0.25, 0.50, and 1.0 mg/L. The matrix effect caused by other components in the sample matrix will interfere with the ionization of target compounds when using an ESI ion source and then affect the accuracy of quantitative analysis method [10,11]. The matrix effect (ME%) was calculated as follows [12]:

(1) $$\mathrm{ME}\%=\frac{m_{matrix}-m_{solvent}}{m_{solvent}}\times 100\%$$

where $m_{matrix}$ and $m_{solvent}$ are the slope of calibration curves in matrix and slope of calibration curves in the solvent, separately.

### 2.5. Dissipation of Benziothiazolinone

The dissipation curve of benziothiazolinone was fitted by the first-order rate equation:

(2) $$C_{t}=C_{0}\times exp\left(-kt\right)$$

where $C_{0}$ and $C_{t}$ are represented sample initial concentration (mg/kg) and residue concentration (mg/kg) at time *t* (d). And the half-life of degradation (*DT*_50_) was calculated using Hoskins’ formula (*DT*_50_ *=* ln2/*k*).

### 2.6. Chronic Dietary Risk Assessment

National estimated daily intake (NEDI) for long-term intake risk and the risk quotient (RQ) were calculated by the following formulas [13]:

(3) $$\mathrm{NEDI}=\sum {\mathrm{STMR}}_{i}\times F_{i}$$

(4) $$\mathrm{RQ}=\mathrm{NEDI}/\left(\mathrm{ADI}\times \mathrm{bw}\right)\times 100\%$$

where $S{\mathrm{TMR}}_{i}$ (mg/kg) was the supervised trials median residue of benziothiazolinone in apple in China. If there was no suitable ${\mathrm{STMR}}_{i}$, the corresponding MRLs could be used instead of calculating the NEDI value. $F_{i}$ (kg) was the average daily intake of a certain food in China; bw was the average weight of Chinese adults (63 kg).

### 2.7. Field Trials

The open-field trials were designed in accordance with the Guideline on Pesticide Residue Trials (NY/T 788–2018) published by the Ministry of Agriculture and Rural Affairs, P. R. China. Field trials were implemented in 8 different sites in China, include Huaibei, Anhui (116.87 E, 34.07 N, semi-humid monsoon climate zone of warm temperate zone), Yinchuan, Ningxia (106.19 E, 38.57 N, temperate continental arid and semi-arid climate), Liaoyang, Liaoning (123.14 E, 41.27 N, temperate continental monsoon climate), Jinzhong, Shanxi (112.69 E, 37.54 N, temperate continental monsoon climate), Baoji, Shaanxi (107.18 E, 34.65 N, warm temperate monsoon climate), Xinxiang, Henan (113.71 E, 35.00 N, temperate continental monsoon climate), Jinan, Shandong (117.21 E, 36.82 N, temperate continental monsoon climate), Kunming, Yunnan (102.52 E, 25.19 N, the mountain monsoon climate of the low latitude plateau in the North subtropics). Each experimental plot was designed to contain at least 4 apple trees, and protective belts were set between the plots. Furthermore, an experimental control plot (no less than 4 apple trees) was implemented at each treatment level.

For the final residues experiments on apple, 2% benziothiazolinone suspension concentrate (SC) was diluted with water and sprayed 3 times on experimental plots at the maximum recommended dosage of 25 mg a.i./kg. Each spraying interval was 7 days. The blank control groups were not sprayed with pesticide. For experimental plots, the samples of apple fruits with normal growth, disease-free, and mature were collected from no less than 4 fruit trees at 14, 21, 28 days after the last spraying by the random method and packed in plastic bags. Two independent samples were collected at each time. For blank control groups, the samples were collected by the same method at 14 and 28 days after the last spraying.

Two percent of benziothiazolinone SC was applied at the dosage of 37.5 mg a.i./kg, treatment samples of apple fruits were collected randomly from each plot at 0 (2 h),1,3, 7, 10, 14, 21, 30, 45 days after the last application and the control samples were collected at 0 (2 h) and 35 days to evaluate the dissipation kinetics of benziothiazolinone. All samples were chopped, mixed, divided into 2 subsamples of 150 g, and then labeled and stored at −20 °C in the dark (polyethylene bags) for further analysis.

## 3. Results

### 3.1. Optimization of Extraction and Purification

Currently, there are relatively few studies on the residues of benziothiazolinone. In previous studies, the extraction effects of acetonitrile, acetonitrile containing 0.1% acetic acid, n-hexane, ethyl acetate, and acetone on benziothiazolinone with an apple as analysis sample were compared. Acetonitrile and 0.1% acetic acid acetonitrile obtained outstanding recoveries [8]. The recoveries ranged from 77.4% to 84.9% for benziothiazolinone in tobacco powder samples extracted by acetonitrile-water mixed solution [14]. For the citrus samples, four organic solvents (methanol, acetonitrile, ethyl acetate, and dichloromethane) were compared, and acetonitrile supplied outstanding extraction efficiencies for benziothiazolinone [9]. Thus, acetonitrile was used as the extraction solvent of benziothiazolinone in this study.

Various solid sorbents, including PSA, GCB, and C_18_, are widely used to remove co-extractives and interferences, which can improve the signal-to-noise ratio (S/N) of target analytes [15,16]. Three sorbents of PSA, C_18_, and GCB were investigated via recovery tests to obtain satisfactory cleanup in pretreatment. As is well known, PSA is normally applied to remove matrix compounds, such as sugar and fatty acids. Conversely, C_18_ is commonly used for antiphase extraction [17]. The GCB sorbent is known for its ability to remove pigments efficiently, especially chlorophyll. The results showed that the recoveries of benziothiazolinone were all less than 70% when the amount of the sorbents was 50 mg PSA or GCB. When 50 mg C_18_ was used, the recovery and RSD were satisfactory. These results are consistent with the feature of C_18_ suitable for extracting non-polar and medium-polar compounds from polar samples [18]. Therefore, 50 mg C_18_ was applied to purify the extract acetonitrile phase in our work.

### 3.2. Method Validation

Based on the Guideline on Pesticide Residue Trials, the analytical method was validated in terms of linearity, sensitivity, precision, accuracy, and matrix effect. In this study, the matrix-matched standard calibrations were used to eliminate the matrix effect [19,20]. The benziothiazolinone standard solution was diluted with apple blank matrix extract to prepare 0.010, 0.025, 0.050, 0.10, 0.25, 0.50, 1.0 mg/L standard solutions, and the determination was carried out under the above instrument conditions. According to the above method, the standard samples with seven concentration levels between 0.01 and 1 mg/L diluted by acetonitrile solvent and blank matrix were analyzed, respectively, using three replicates of each concentration level to obtain the equation for the average calibration curves. The matrix-matched average calibration curve (y=25241x−245.85, where x and y were defined as the standard solution injection concentration and average peak area of quantitative ion, respectively) and the standard solution average calibration curve (y=25795x+5.0972) were obtained separately with the correlation coefficients (r) higher than 0.9995. The calculated result of the matrix effect was −2.1%, demonstrating that the matrix of apple samples exerted a weak inhibitory effect on benziothiazolinone.

The accuracy and precision of the method were verified by five repeated recovery experiments at three spiked concentrations. As shown in Table 1, the average recoveries of benziothiazolinone in the apple matrix were 85.5–100.2% with the RSD of 0.8–14.9%, indicating the present method has great accuracy and precision.

**Figure 2:**
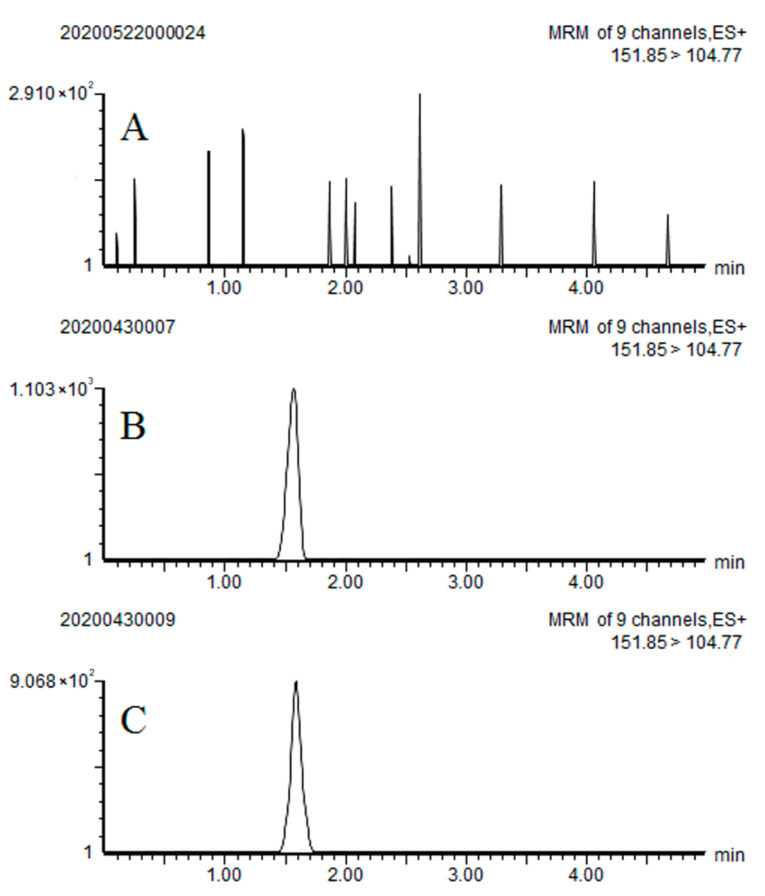
Typical liquid chromatography tandem mass spectrometry/multiple reaction monitoring LC-MS/MS MRM chromatograms of benziothiazolinone in apple samples: (**A**) blank sample; (**B**) matrix standard at 0.01 mg/kg; and (**C**) spiked sample at 0.01 mg/kg.

The limit of quantification (LOQ) was defined as the minimum spiked concentrations of target analytes in the matrix. According to the recovery experiments, under the above analysis conditions, the limit of quantification of benziothiazolinone in apple was 0.01 mg/kg. Typical LC-MS/MS chromatograms of benziothiazolinone are shown in Figure 2. The above validation results suggested that the analytical method was reliable for the determination of benziothiazolinone residue in apple.

### 3.3. Dissipation Behaviors

**Figure 3:**
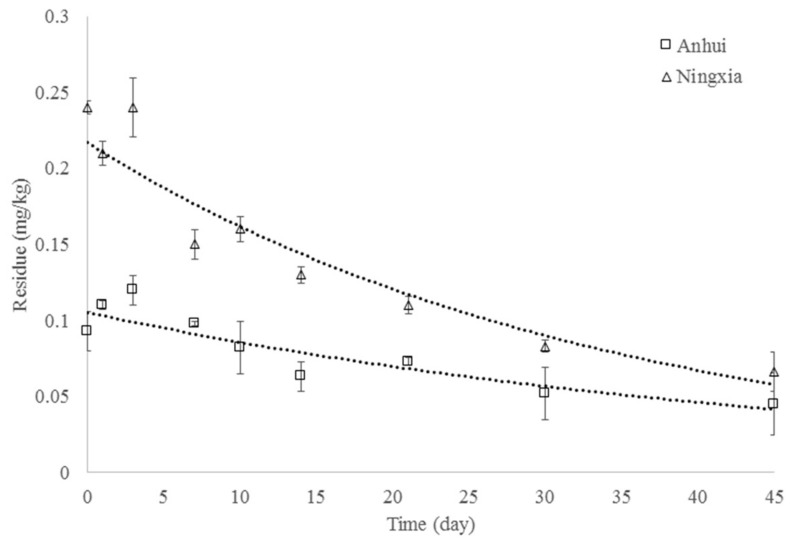
Dissipation curves (*n* = 3) of benziothiazolinone in apple in Anhui and Ningxia.

The degradation dynamics and dissipation curves (*n* = 3) of benziothiazolinone in apple in Anhui and Ningxia are presented in Figure 3. The residue of benziothiazolinone in apple gradually decreased with time, and the dissipation process was of first-order reaction kinetics [21,22,23]. The initial concentrations of benziothiazolinone at 0 days (2 h after application) in apple were low both in Anhui and Ningxia, and the degradation half-lives of benziothiazolinone were 33.0 days and 23.9 days with dissipation equations of $C=0.1053e^{-0.021t}$ and $C=0.2168e^{-0.029t}$, respectively. Different dissipation rates and initial residue may be caused by environmental factors, including climate type and soil type at different sites [24,25].

### 3.4. Final Residue

As shown in Table S3, the results of quality control (QC) indicated that the average recoveries of benziothiazolinone in apple were 89.7–95.9% with the RSD of 3.5–4.8%. These data indicate that the detection method of benziothiazolinone is stable and accurate, and the detection of field trial samples was reliable.

Final residues were investigated by analyzing apples sampled at three time intervals after spraying. As shown in Table 2, after applying 2% benziothiazolinone SC three times with intervals of 7 days, the final residues of benziothiazolinone in apple were less than 0.01 mg/kg in all samples. Therefore, at the pre-harvest interval (PHI) of 14 days, the final residues of benziothiazolinone in apple were lower than the MRL of 0.05 mg/kg set by China. These dates will provide a reference for benziothiazolinone’s proper use in apple.

### 3.5. Dietary Risk Assessment

According to the dietary structure of China and the supervised trial median residues (STMRs) or MRLs of crops registered to use benziothiazolinone, the NEDI value was calculated, and the RQ was obtained by comparing with the ADI value of benziothiazolinone to assess the dietary risk. The ADI value of benziothiazolinone was 0.017 mg/kg bw from GB 2763-2019 [6]. The calculation results are shown in Table 3.

As displayed in Table 3, the RQ (15.5%) of benziothiazolinone was less than 100%, manifesting that the application of benziothiazolinone in apple with recommended dosage will not bring about risk for common Chinese consumers.

## 4. Conclusions

In this study, a sensitive and effective QuEChERS-LC-MS/MS method was constructed to detect the residues of benziothiazolinone in apple. The results of field trials in eight locations showed that the final residues of benziothiazolinone in all samples were less than LOQ 0.01 mg/kg and below the MRL 0.05 mg/kg recommended by China. Although the degradation half-lives of benziothiazolinone were 23.9–33.0 days, the RQ value of dietary risk assessment was 15.5%, which suggested that the application of 2% benziothiazolinone SC at the recommended dosage would be safe for consumers. As the first independently developed fungicide in China, it will be registered in different countries in the future. This study could provide an effective analytical method for the detection of benziothiazolinone in apple and provide guidance for dietary risk assessment of benziothiazolinone residue in apple. However, considering that benziothiazolinone may cause potential health risks to consumers, the more toxicological studies of benziothiazolinone are needed. In addition, due to its longer half-life in apple, the metabolic pathway and ecological risk of benziothiazolinone also need to be further studied.

## Figures and Tables

**Table 1:** Recoveries of benziothiazolinone in apple.

Compound	Spiked Level (mg/kg)	Recoveries (%)						RSD (%)
		1	2	3	4	5	Average Value	
benziothiazolinone	0.01	78.9	70.7	94.9	80.8	102.0	85.5	14.9
	0.05	105.4	87.8	101.7	102.3	104.0	100.2	7.1
	1.0	98.5	99.3	100.3	100.5	99.0	99.5	0.8

**Table 2:** Final residues of benziothiazolinone in apple samples.

Location	Dosage (mg a.i./kg)	Spray Times	Intervals (d)	Final Residue (mg/kg)	STMR * (mg/kg)	HR ** (mg/kg)
Liaoning, Henan, Shaanxi, Shanxi, Shandong, Yunnan, Anhui, Ningxia	25	3	14	<0.01	<0.01	<0.01
			21	<0.01	<0.01	<0.01
			28	<0.01	<0.01	<0.01

* SMTR: supervised trial median residue; ** HR: highest residue.

**Table 3:** Dietary risk assessment of benziothiazolinone in apple.

Food Classification	Fi (kg)	Reference Residue Limits (mg kg^−1^)	Sources	NEDI (mg)	ADI (mg)	Risk Probability (%)
Rice and its products	0.2399	0.05	China	0.1200	0.017 × 63	
Flour and its products	0.1385	0.2	China	0.0277		
Other cereals	0.0233					
Tubers	0.0495					
Dried beans and their products	0.016					
Dark vegetables	0.0915					
Light vegetable	0.1837	0.1	China	0.01837		
Pickles	0.0103					
Fruits	0.0457	0.01	STMR	0.000457		
Nuts	0.0039					
Livestock and poultry	0.0795					
Milk and its products	0.0263					
Egg and its products	0.0236					
Fish and shrimp	0.0301					
Vegetable oil	0.0327					
Animal oil	0.0087					
Sugar, starch	0.0044					
Salt	0.012					
Soy sauce	0.009					
Total	1.0286			0.1665	1.071	15.5

## Data Availability

The data presented in this study are available on request from the corresponding author. The data are not publicly available due to privacy.

## Supplementary Materials

The following are available online at https://www.mdpi.com/article/10.3390/ijerph18094478/s1, Table S1: Gradient elution procedure, Table S2: Experimental parameters and chromatographic conditions of benziothiazolinone, Table S3: Quality control (QC) of real sample detection.

## References

[1] Liu H.J., Guo B.Y., Wang H.L., Li J.Z., Zheng L. (2014). Determination of Bromothalonil Residues and Degradation in Apple and Soil by QuEChERS and GC-MS/MS. Bull. Environ. Contam. Toxicol..

[2] Tang W., Ding Z., Zhou Z.Q., Wang Y.Z., Guo L.Y. (2012). Phylogenetic and Pathogenic Analyses Show That the Causal Agent of Apple Ring Rot in China Is Botryosphaeria dothidea. Plant Dis..

[3] Yu F., Liu W., Qi L. (2009). Residue Analysis of Benziothiazolinone in Cucumber and Soil. Agrochemicals.

[4] He L., Mao Y. (2009). Determination of Lead Content in BIT by Flame Atomic Absorption Spectrophotometry. Stud. Trace Elem. Health.

[5] Edwards S.G., Seddon B. (2001). Mode of antagonism of Brevibacillus brevis against Botrytis cinerea in vitro. J. Appl. Microbiol..

[6] National Food Safety Standard Maximum Residue Limits for Pesticides in Food (2019). GB 2763-2019.

[7] Zhao Z., Ge Q., Niu Y., Zhang F. (2015). Determination of Benziothiazolinone Residues in Wheat and Soil by HPLC. Agrochemicals.

[8] Su Y., Zhou J., Meng R., Li N., Li Y., Luo Q. (2016). Determination of benziothiazolinone residue in vegetable and fruit by QuEChERS-liquid chromatography-tandem mass spectrometry. Chin. J. Pestic. Sci..

[9] Liu X.W., Song B.Y., Jiang X.X., Yang Y., Lu P., Hu D.Y. (2021). Residue determination and risk assessment of benziothiazolinone in citrus by LC-MS/MS. Int. J. Environ. Anal. Chem..

[10] Netto P.T., Teixeira Junior O.J., Viana de Camargo J.L., Ribeiro M.L., Rodrigues de Marchi M.R. (2012). A rapid, environmentally friendly, and reliable method for pesticide analysis in high-fat samples. Talanta.

[11] Yu W.W., Huang M., Chen J.J., Wu S.Z., Zheng K.M., Zeng S., Zhang K.K., Hu D.Y. (2017). Risk assessment and monitoring of dinotefuran and its metabolites for Chinese consumption of apples. Environ. Monit. Assess..

[12] Matuszewski B.K., Constanzer M.L., Chavez-Eng C.M. (2003). Strategies for the assessment of matrix effect in quantitative bioanalytical methods based on HPLC-MS/MS. Anal. Chem..

[13] Sun M.N., Tong Z., Dong X., Chu Y., Wang M., Gao T.C., Duan J.S. (2019). Determination of the Residue Behavior and Risk Assessment of Chlorfluazuron in Chinese Cabbage, Kale, Lettuce and Cauliflower by UPLC-MS/MS. Int. J. Envion. Res. Public Health.

[14] Long S., Wu Y., Zhang N., Li W., Hu C. (2019). Residue and Dissipation Dynamics of Benziothiazolinone on Tobacco. Guizhou Agric. Sci..

[15] Walorczyk S., Drozdzynski D., Kierzek R. (2015). Determination of pesticide residues in samples of green minor crops by gas chromatography and ultra performance liquid chromatography coupled to tandem quadrupole mass spectrometry. Talanta.

[16] Li C.D., Liu R., Li L., Li W., He Y.J., Yuan L.F. (2017). Dissipation behavior and risk assessment of butralin in soybean and soil under field conditions. Environ. Monit. Assess..

[17] Li C., Chen Z., Qin D., Liu R., Li L., Li W., He Y., Yuan L. (2020). Simultaneous determination of the herbicide bixlozone and its metabolites in plant and animal samples by liquid chromatography-tandem mass spectrometry. J. Sep. Sci..

[18] Zhao Z., Sun R., Su Y., Hu J., Liu X. (2021). Fate, residues and dietary risk assessment of the fungicides epoxiconazole and pyraclostrobin in wheat in twelve different regions, China. Ecotox. Environ. Safe.

[19] Rutkowska E., Lozowicka B., Kaczynski P. (2019). Three approaches to minimize matrix effects in residue analysis of multiclass pesticides in dried complex matrices using gas chromatography tandem mass spectrometry. Food Chem..

[20] Besil N., Cesio V., Heinzen H., Fernandez-Alba A.R. (2017). Matrix Effects and Interferences of Different Citrus Fruit Coextractives in Pesticide Residue Analysis Using Ultrahigh-Performance Liquid Chromatography-High-Resolution Mass Spectrometry. J. Agric. Food Chem..

[21] Huang Y.H., Lin Z.Q., Zhang W.P., Pang S.M., Bhatt P., Rene E.R., Kumar A.J., Chen S.H. (2020). New Insights into the Microbial Degradation of D-Cyphenothrin in Contaminated Water/Soil Environments. Microorganisms.

[22] Bhatt P., Zhang W.P., Lin Z.Q., Pang S.M., Huang Y.H., Chen S.H. (2020). Biodegradation of Allethrin by a Novel Fungus Fusarium proliferatum Strain CF2, Isolated from Contaminated Soils. Microorganisms.

[23] Feng Y.M., Zhang W.P., Pang S.M., Lin Z.Q., Zhang Y.M., Huang Y.H., Bhatt P., Chen S.H. (2020). Kinetics and New Mechanism of Azoxystrobin Biodegradation by an Ochrobactrum anthropi Strain SH14. Microorganisms.

[24] Paramasivam M. (2021). Dissipation kinetics, dietary and ecological risk assessment of chlorantraniliprole residue in/on tomato and soil using GC-MS. J. Food Sci. Technol. Mysore.

[25] Kalsi N.K., Kaur P. (2019). Dissipation of bispyribac sodium in aridisols: Impact of soil type, moisture and temperature. Ecotox. Environ. Safe.

