# Supplementary material for: Dissipation Behavior, Residue, and Risk Assessment of Benziothiazolinone in Apples
Source: Int J Environ Res Public Health. 2021 Apr 23;18(9):4478. doi: 10.3390/ijerph18094478 (PMC8122877; doi:10.3390/ijerph18094478)
Supplement: Supplementary file 1 [file ijerph-18-04478-s001.zip › ijerph-1131510-SI.pdf]

Table S1. Gradient elution procedure.

t/min	flow rate(ml/min)	Methanol (%)	0.05%formic acid aqueous solution (%)
0	0.40	12.0	88.0
1.0	0.40	98.0	2.0
3.9	0.40	98.0	2.0
4.0	0.40	12.0	88.0
5.0	0.40	12.0	88.0

Table S2. Experimental parameters and chromatographic conditions of benziothiazolinone

Compound	Molecular formula	Precursor ion (m/z)	RT (min)	Ion source	CV (V)	Quantification ion transition (m/z)	CE1 (eV)	Confirmatory transition (m/z)	CE2 (eV)	Ion ratio
Benziothiazolinone	C_7H_5SON	151.18	1.57	ESI+	52	151.85/104.77	20	151.85/76.80	28	1.5

RT, retention time; CV, cone voltage; CE, collision energy

Table S3. Quality control (QC) of benziothiazolinone in real sample detection.

Matrix	Spiked level (mg/kg)	Date of QC detected	Recoveries (%)					
			1	2	3	4	Average	RSD (%)
apple	0.10	13/5/2020	91.9	94.3	99.1	98.2	95.9	3.5
		19/5/2020	90.0	94.0	83.8	90.9	89.7	4.8
		22/5/2020	96.5	99.0	88.6	95.2	94.8	4.7
